# Therapeutic Notes

**Published:** 1892-09

**Authors:** William C. Krauss

**Affiliations:** Professor of Pathology, Niagara University, Medical College


### THERAPEUTIC NOTES.

By WILLIAM C. KRAUSS, M. D.,

Professor of Pathology, Niagara University, Medical College.

### TREATMENT OF CHOREA BY EXALGINE.

In the *Journal of Nervous and Mental Disease* for July, 1892, Dr. Charles L. Dana, of New York, gives the results obtained in sixteen cases of chorea treated with exalgine. He expresses his opinion thus: "I have seen such results from exalgine properly given as to convince me that it unquestionably has a specific effect on the ordinary or Sydenham's chorea." His method of administration is by means of two-grain capsules, one three times a day the first day; one four times the second day; one five i. d. the third day; finally, three grains five i. d., if needed. The indications for its use are the common subacute chorea of Sydenham, not chronic chorea, habit chorea, convulsivitic, or chorea major. He has abandoned exalgine for the relief of pain, not having been rewarded with anything like favorable results.

[The abstractor has tried to use exalgine for the relief of pain in nervous diseases, also in two cases of chorea of Sydenham. His results were as discouraging and painful to the patient as to himself, and the drug was abandoned.]

### THE TREATMENT OF INSOMNIA.

Dr. Joseph Collins, of New York, in an interesting article on insomnia, published in the *Journal of Nervous and Mental Disease*, July, 1892, contrasts the action of chloralamid and sulfonal in the treatment of insomnia, and arrives at the following conclusions :

1. Chloralamid is a safe and one of the most reliable hypnotics.

2. It is not ordinarily followed by distressing after-symptoms, particularly headache.

3. It is especially valuable as a hypnotic where pain is a prominent factor, but not violent.

4. In cases of insomnia, where there is excessive activity of the brain, it is also useful.

5. On account of its stimulating activity on the respiratory function, it is the hypnotic *par excellence* in nervous exhaustion, associated with an asthenic condition of respiration and symptom complex indirectly dependent on this, brought about by defective oxidation and the formation of unstaple chemical compounds in the system.

6. On account of its very slight action in depressing the circulation, it can be given in diseases associated with a weak heart, with greater safety than most of the other hypnotics, not excepting chloral itself.

7. It is conveniently administered in the shape of an elixir, and this overcomes the need of dissolving it.

8. Its dose is from one to three scruples, administered one hour before sleep is desired, and this should not be repeated within two hours, for occasionally the action of the drug is delayed.

Sulfonal is preferred when we wish to get very rapid action. It should be given dissolved in boiling water, taken as hot as possible. In this way it is at once absorbed, sleep frequently occurring in from fifteen to twenty minutes. The disadvantages of sulfonal are that the patient is liable to form the sulfonal habit, and that its effects last through part of the following day.

---

### HYPNOTIC SUGGESTION FOR IMPOTENCE.

Impotence, according to Gywrkovecky, may be cured in its varied forms by hypnotic suggestion, which he has also found a very useful means of treatment in masturbation, nocturnal pollutions, etc.

In three cases of sexual neurasthenia he has had complete success from suspension in the Sayre apparatus.—*Wiener Med. Presse.*—*Medical Age.*

---

### ON THE EMPLOYMENT OF SALANINE IN AFFECTIONS OF THE STOMACH, ESPECIALLY GASTRALGIA.

Dr. Sesnos, of Paris, has lately taken up the investigation of the effects of salanine in seventeen cases of gastralgia. Of this number, four were unsuccessful and thirteen successful. Of the successful cases, several were of gastric ulcers which had resisted all other forms of treatment. One case of cancer of the pylorus, with severe pain, vomiting, sleeplessness, etc., yielded to the drug, and marked improvement in all the symptoms was noticed. After continuing to progress favorably for several days, the patient was taken from the hospital by his friends, much improved in every condition.

The dose of salanine given daily was 5–15 centigrammes, in pill form, generally one hour before meals. As a rule, the administration of the drug was followed by no unpleasant symptoms.—*Bulletin Général de Thérapeutique*, June 30, 1892.

---

### THE EFFECT OF PILOCARPIN UPON EPILEPTICS.

Dr. C. Féré, of Paris, was led to make a series of investigations on the use of pilocarpin in epilepsy, by reading the report of a Russian who has succeeded in treating cases in this manner. Dr. Féré's results were far from satisfactory. The injections of very small quantities were followed by the symptoms of an approaching attack, and, after a brief interval, the attack itself. In one case of epilepsy, in which the attacks had disappeared for some time, the injections called forth new attacks. Dr. Féré advises not to administer it in epilepsy, and to advoid it in old epileptics who have stopped the use of the bromides.—*Ext. des Comptes rendu des Séances de la Société de Biologie.*

---

### ARSENIC IN CHOREA.

In a case of chorea treated by the abstractor, a girl of thirteen years, arsenic was cautiously administered at the onset of the affection, and the dose gradually increased until thirty-three drops of Fowler's solution was taken three times daily. At no time did any toxic symptoms of the stomach or perpheral nerves arise, and, furthermore, no beneficial results could be detected.

